# Pilot study: transduction of primary paraganglioma chromaffin tumour cells with inducible c-MYC drives cell proliferation

**DOI:** 10.1055/a-2797-3576

**Published:** 2026-03-25

**Authors:** Juan Zhang, Heggert G. Rebel, Dominique Duesman, Charlotte J. Dommering, Abbey Schepers, Peter Devilee, Jean-Pierre Bayley

**Affiliations:** 1 4501 Human Genetics, Leiden University Medical Center, Leiden, Netherlands; 2 1209 Human Genetics, Amsterdam UMC Location VUmc, Amsterdam, Netherlands; 3 4501 Department of Internal Medicine, Leiden University Medical Center, Leiden, Netherlands

**Keywords:** cell model, SDHB, SDHD, paraganglioma, pheochromocytoma, MYC

## Abstract

Succinate dehydrogenase (SDH) gene variants are the most common cause of the neuroendocrine tumour hereditary paraganglioma, which is associated with an over 20% metastasis risk as well as significant morbidity. There are currently no relevant human tumour cell lines or mouse models, and molecular understanding of downstream tumourigenic pathways is still rudimentary despite over two decades of concerted effort worldwide. These tumours generally show extremely slow in vivo doubling times (4–12 years), presumably existing in a primarily semi-quiescent state with little cell cycling or DNA replication. This characteristic makes deriving a useful tumour cell line impractical. A better alternative would be a cell line in which cell proliferation can be turned on and off at will, allowing expansion to generate sufficient cell numbers and experimentation once tumour cells have returned to their natural semi-quiescent state. The closest models currently available, highly-proliferating rat and mouse adrenal paraganglioma cell lines, are molecularly unrelated to SDH tumours. In this pilot study, we investigated whether primary SDH-derived paraganglioma tumour cells can be made to proliferate in vitro. We successfully transduced primary paraganglioma tumour cells with a lentiviral construct, using the proven strategy of c-MYC¬T58A (c-MYC) controlled by a Tet-On doxycycline-inducible expression system. We present the first evidence that primary paraganglioma chromaffin cells can be induced to proliferate in vitro, even in later passage cultures. Without any prior selection for chromaffin tumour cells, passaged cultures were obtained with over 80% synaptophysin-expressing chromaffin tumour cells, suggesting that this highly promising strategy deserves further exploration.

## Introduction

Paragangliomas (PGLs) are highly vascular neuroendocrine tumours that originate from specialized neurosecretory cell clusters with a variety of functions such as monitoring changes in the levels of oxygen and CO _2_ or the excretion of catecholamines. Parasympathetic PGLs arise in the parasympathetic ganglia of the head and neck (HNPGLs), approximately 60% of which are found in the carotid body, while the remaining 40% occur in the vagal body and jugulotympanic paraganglia 1 . Sympathetic paragangliomas (PPGLs) are most commonly found within the adrenal medulla (formerly termed pheochromocytomas but according to the 5th WHO Classification of Endocrine and Neuroendocrine Tumors 2022 should now be described as adrenal paragangliomas 2 ), but also arise along the sympathetic nervous system between the aortic arch and urinary bladder (sympathetic paragangliomas).

Causative or suspected causative germline variants are found in around 25–40% of all PPGLs/HNPGLS 3 4 5 6 . These include cancer syndrome-associated genes such as *RET, VHL* , or *NF1* , together with a diverse range of genes with metabolic ( *SDHA, SDHB, SDHC, SDHD, SDHAF2, FH, MDH2, IDH1, IDH2, DLST, SLC25A11, SUCLG2, GOT2* ) or diverse functions ( *HRAS, EPAS1* [HIF2A], *TMEM127 and MAX* ) 7 . Of the metabolism-associated genes, those encoding subunits of succinate dehydrogenase (SDH) are the most clinically important in PPGL/HNPGL. Succinate dehydrogenase, an enzyme also acting as mitochondrial complex II, is located in the inner mitochondrial membrane where the catalytic subunit SDHA converts succinate to fumarate as part of the tricarboxylic cycle, releasing electrons that are transported by subunit B (SDHB) via reductive iron-sulphur centres to the membrane-spanning subunits C and D (SDHC and SDHD), where they reduce the mobile electron transporter ubiquinone to ubiquinol.

Paragangliomas are typically slow-growing masses, with estimated tumour doubling times of between 4 and 12 years 8 9 , a characteristic underlined by the less than 1% proliferative activity typically found with Ki-67 immunostaining 10 . Accordingly, patient survival rates are relatively good and even patients with metastatic tumours show a 5-year overall survival of 76–85% 11 12 13 . Nonetheless, metastasis is relatively common (approx. 20%) in 14 15 and morbidity due to hypertension 16 , invasive tumour growth and surgical complications can be significant 17 .

Surgical resection is currently the standard treatment, with options for radio- and chemotherapy, as well as emerging options for targeted therapies such as the tyrosine kinase inhibitor sunitinib and the HIF2α inhibitor belzutifan 18 .

An improved understanding of other targetable molecular pathways will require appropriate mouse and cell models, as current models are either unrelated to SDH-associated disease or have other weaknesses. For example, the widely used genetically-modified adrenal paraganglioma mouse cell lines, MPC and MTT, are based on an *NF1* knockout background and irradiated. Together with knockdown or knockout of an SDH subunit gene they effectively mix at least two, and most likely three or more distinct PGL drivers. Another mouse cell line, dubbed ‘immortalized mouse chromaffin cells’ (imCC), was derived from an Sdhb knockout mouse, but has never been satisfactorily characterized 19 . The human cell line hPheo1, derived from a sporadic adrenal pheochromocytoma, and originally referred to as a “progenitor cell line” 20 , was immortalized using hTERT and subjected to a neuronal differentiation regime consisting of BMP4, NGF and dexamethasone. This cell line has not been shown to derive from chromaffin tumours cells and no evidence has been presented to show that it is tumorigenic. Only one SDH-related (rat-derived adrenal paraganglioma) tumour cell line has been developed to date, which also required initial radiation to induce tumour formation 21 . For a detailed overview of available cell lines, see Bayley and Devilee 2020 22 .

A human paraganglioma-adrenal paraganglioma cell culture model would not only allow investigation of the origin and targetable vulnerabilities of human PGLs, it could also serve as a valid platform to test potential therapeutics. The difficulty of generating a human PGL cell line using conventional approaches was underlined by a study from our group in which we cultured over 60 primary HNPGLs/PPGLs without successfully deriving a stable cell line 23 .

In view of the extremely slow growth of paragangliomas, inducible gene expression systems are an attractive option for maintaining and expanding the tumour cell fraction of primary cultures. These systems permit modulation of (trans)gene expression, ideally allowing a gene of interest to be controlled in a quantitative manner via an effector substance [e. g. tetracycline or derivatives such as doxycycline (dox)] that activates gene expression in a reversible, dose-dependent fashion. The doxycycline-inducible c-MYC transgene (Tet-On-MYC) construct used in the present study has previously been successfully used to control c-MYC expression during differentiation of ventricular-like cells, pacemaker-like cells and smooth muscle cells, mimicking the cardiac developmental program 24 . c-MYC is a transcription factor and proto-oncogene that regulates the expression of genes involved in cell proliferation, apoptosis and differentiation. In addition, c-MYC influences metabolism by upregulating proteins involved in glycolysis, including GLUT1, hexokinase, phosphofructokinase and lactate dehydrogenase A, as well as via mitochondrial biogenesis and glutamine catabolism, all of which have been implicated in the tumourigenesis of PGLs 25 26 . Phosphorylation of c-MYC at threonine 58 (T58A) increases protein stability, an effect partially mimicked in the c-MYC ^T58A^ mutant 27 .

In this pilot study we investigated whether primary paraganglioma chromaffin cells can be successfully transduced with lentiviral constructs, if expression of an introduced copy of the c-MYC ^58A^ -protein can be controlled using a Tet-On doxycycline-inducible expression system and whether this expression induces the proliferation of chromaffin cells.

## Materials and methods

See **appendix 1** .

## Results

### Lentiviral transduction

**Fig. 1:**
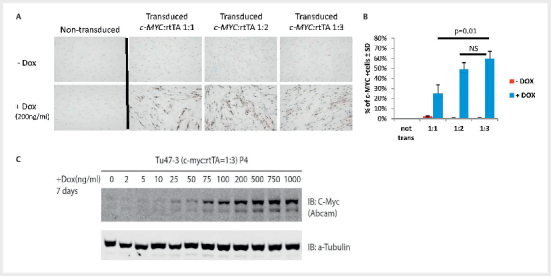
Optimization of transduction with c-MYCT58A/rtTA plasmids in cell cultures derived from a primary carotid body paraganglioma (Tu47). ( **A** ) Tumour cultures were transduced with lentiviruses in several different ratios and cultured with or without doxycycline (dox) (200ng/ml). ( **B** ) Quantification of panel A. ( **C** ) Western blot with immunostaining for c-MYC, with a-Tubulin as loading control in Tu-47, culture P4. The c-MYCT58A and rtTA plasmids were transduced at a 1:3 ratio and cells were subsequently cultured with a single dose of doxycycline, in a range of concentrations, for 7 days before fixation and protein analysis.

We first determined whether cells derived from a primary tumour culture (Tu47, a carotid body tumour carrying the SDHD variant p.(Asp92Tyr)) could be successfully transduced with lentivirus containing c-MYC and rtTA. To establish the optimal ratio of c-MYC to rtTA, we used the vectors in several different ratios. Non-transduced Tu47 cells cultured with or without doxycycline were completely negative upon c-MYC staining ( Fig. 1A , upper left panel). Transduced cells not exposed to doxycycline showed very low levels of c-MYC protein expression ( Fig. 1A , three upper panels right). By contrast, addition of doxycycline induced c-MYC in around 60% of transduced Tu47 cells ( Fig. 1A , three lower panels right) and resulted in increasingly tightly-packed, highly dense cell cultures with elevated c-MYC:rtTA ratios ( Fig. 1B , p=0.01, all experiments in triplicate). The dose-dependent inducibility of c-MYC was also confirmed by western blot ( Fig. 1C ). The optimal ratio was determined as 1:3.

### Transducing primary tumour cell cultures

**Fig. 2:**
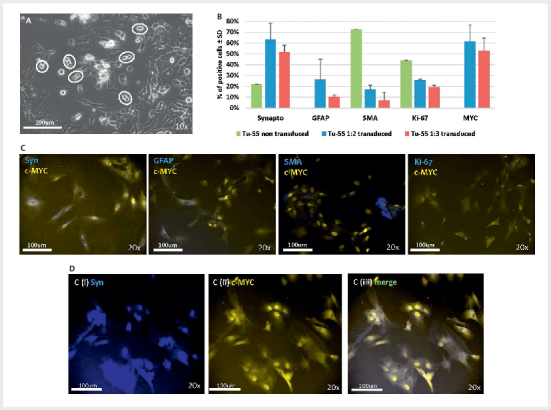
A carotid body tumour culture (Tu55) transduced with c-MYC ^T58A^ displays significantly enhanced numbers of synaptophysin+ (marker for chromaffin cells) and GFAP+ (marker for sustentacular) cells, as well as lower levels of SMA+ fibroblasts. ( **A** ) Phase contrast microscopy revealed numerous mitotic figures (white ovals), suggesting high rates of cell proliferation. Similar figures were not seen in any non-transduced tumour culture over a two-year period. ( **B** ) Antibody staining of non-transduced and transduced Tu-55 (1:2 transduction or 1:3 transduction ratios, in duplicate) with a range of cell-specific and general marker proteins 12 days after transduction. Experiments in duplicate, therefore not statistically significant. ( **C** ) Representative images for ( **B** ). (i) Tu-55 1–2 stained for c-MYC (yellow) and synaptophysin (blue) (ii) Tu-55 1–3 stained for c-MYC (yellow) and GFAP (blue), (iii) HR Tu-55 1–3 transduced stained for c-MYC (yellow) and SMA (blue), (iv) Tu-55 1–3 stained for c-MYC (yellow) and Ki-67 (blue). ( **D** ) Transduced c-MYC is expressed in synaptophysin-positive cells. (i) Immunofluorescent staining of synaptophysin reveals predominantly cytoplasmic staining, whereas c-MYC (ii) shows strong nuclear staining. (iii) Merging images reveals cellular co-expression.

Having established that our lentiviral system successfully transduces and induces cell division of tumour-derived cells in culture, we obtained a recently operated tumour, tumour 55 (Tu55), a carotid body paraganglioma carrying the SDHB variant p.(Arg217Cys). This culture was transduced with c-MYC:rtTA at the ratios 1:2 and 1:3. The large majority of cells in any freshly cultured PPGL tumour are chromaffin tumour cells, as evidenced by the expression of characteristic immunocytochemical markers, including synaptophysin and neuron-specific enolase 23 . After five days of culture, Tu55 cultures were transduced with lentivirus and distributed across chamber slides. Doxycycline was added to appropriate chamber slides two days after transduction and cells were cultured further. Visual assessment of Tu55 cultures showed frequent mitotic figures ( Fig. 2A ; at least six in a single field), something never previously observed over two years of culturing 62 untransduced PGL cultures 23 .

To determine the relative proportions of cell types, at day 12 after transduction we stained Tu55 cells cultured on chamber slides with or without doxycycline, compared with non-transduced Tu55 cells as a control. Cells were stained with primary antibodies against synaptophysin (a marker identifying chromaffin (tumour) cells), GFAP (sustentacular cells), SMA (fibroblasts/connective tissue), together with Ki-67, a cell proliferation marker, and c-MYC ( Fig. 2B ). Cells expressing synaptophysin or c-MYC were relatively numerous (>60%), as were GFAP-expressing cells (>20%). Levels of Syn+ chromaffin cells were noticeably higher in transduced versus untransduced cultures, while fibroblast numbers (SMA+) were lower in transduced cultures. The overall number of Ki-67+ cells was lower in transduced cultures, but transduced cultures contained 3/4-fold more cells on average, suggesting rapid cell cycling or less apoptosis of primarily synaptophysin+ tumour cells. We then used dual immunofluorescent staining to determine whether synaptophysin-positive cytoplasm co-localized with c-MYC+ nuclei. Dual staining demonstrated successful transduction and induction of c-MYC expression in synaptophysin-expressing cells chromaffin tumour cells ( Fig. 2C ).

### Cell proliferation

**Fig. 3:**
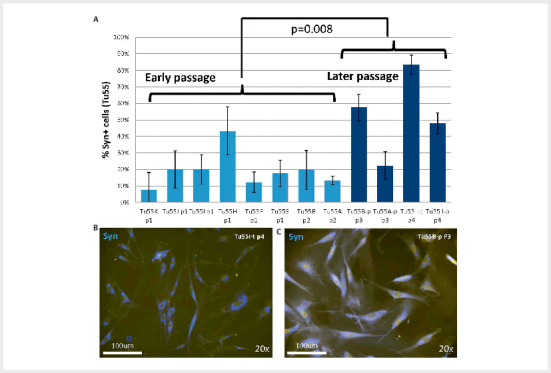
Later passage paraganglioma subclones of Tu55 show significantly enhanced numbers of synaptophysin (Syn+) cells. ( **A** ) A fresh culture of a carotid body paraganglioma (Tu55) was transduced with c-MYCT58A/rtTA and subclones cultured on chamber slides and stained for expression of cell marker proteins as indicated. Subclones showed significantly higher numbers of Syn+ cells in later passages. ( **B&C** ) Representative images of immunofluorescent staining. ( **B** ) Around 20% of cells in Tu55A-p P3 were synaptophysin+ chromaffin cells (7 weeks of culture), while ( **C** ) around 83% of Tu55I-t P4 cells were Syn+ chromaffin cells (8 weeks of culture). (B/I = indicates subclone, p=pipetting, t=trypsin, PX=passage number). When not specifically noted, cells were passaged using trypsin dissociation. All experiments in triplicate.

We then assessed whether transduced cell populations showed evidence of increased proliferation. As no pre-selection method was applied, all cells present in the freshly digested tumour were likely to have been transduced, including chromaffin, sustentacular, neuronal, epithelial, endothelial, immune 28 and connective tissue cells 23 . We have previously shown that the synaptophysin-expressing tumour cell component of PGLs declines rapidly in cell culture. In non-transduced Tu55 cultures synaptophysin-positive cells constituted around 20% of cells by 3 weeks of culture, which is typical for these cultures 23 , whereas transduced Tu55 cultures showed 50–60% Syn+ cells ( Fig. 2B ). Approximately 40% of the cells in the non-transduced Tu55 culture showed evidence of proliferation (Ki-67+) compared to around 20% in the transduced cultures, the former likely attributable to the increasing predominance of readily proliferating SMA+ cells (presumably fibroblasts/connective tissue cells) in non-transduced cultures 23 . We next asked whether synaptophysin-positive cell numbers increase or decline in later passages. Although subcultures of Tu55 showed considerably heterogeneity ( Fig. 3A ), later passage cultures (7–8 weeks) showed significantly more synaptophysin+ cells (p=0.008), indicating increased proliferation and/or improved survival ( Fig. 3B ).

**Fig. 4:**
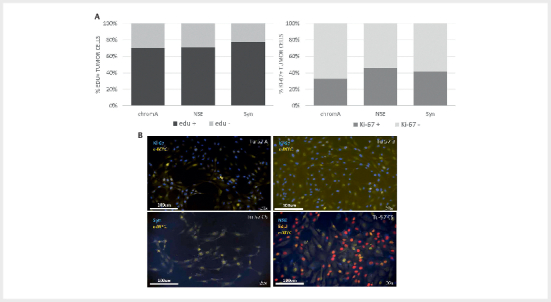
Paraganglioma tumour cells remain highly proliferative at 8 weeks in vitro and co-express chromaffin cell marker proteins. ( **A** ) Quantification of the chromaffin cell markers chromogranin A (CgA), neuron-specific enolase (NSE) and synaptophysin (Syn), together with the markers for cell proliferation EdU and Ki-67. ( **B** ) Representative images of Tu57 subcultures stained for c-MYC and the cell proliferation markers Ki-67-c-MYC or Syn-c-MYC at 4 weeks; or EdU (green nuclei when expressed together) and NSE at 8 weeks of culture.

To replicate the above findings, we acquired a further fresh tumour specimen (Tu57), a carotid body tumour carrying the SDHD Dutch founder variant, p.(Asp92Tyr). Transduced cells of tumour 57 initially showed relatively low levels of synaptophysin+ cells (2–20%), likely due to difficulties digesting the tumour using collagenase B, an issue described previously 23 . We quantified expression of the chromaffin (tumour cell) marker proteins chromogranin A (CgA), neuron-specific enolase (NSE) or synaptophysin (Syn) in individual tumour cells (Tu57 C5, 8 weeks in culture) positive for the proliferation markers EdU (24 hrs), a DNA intercalating dye that indicates cell division over the staining period (in this case 24hrs), or Ki-67, a protein that provides a snapshot of cell division. Cells positive for chromaffin marker proteins showed high proliferation rates, ranging from 70% to 78% in Edu-positive cells and from 33% to 46% in Ki-67-positive cells ( Fig. 4A ). The quantitative differences between Ki-67 and Edu staining indicate an approximate doubling time of 24hrs. Staining for Ki-67 indicated very high rates of cell division in various Tu57 subcultures, including cells expressing synaptophysin or NSE ( Fig. 4B ).

**Fig. 5:**
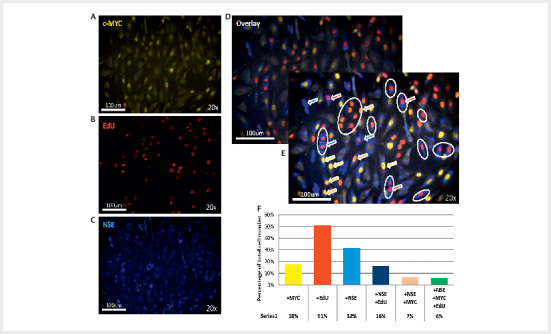
Further evidence that c-MYC ^T58A^ -transduced paraganglioma tumour cells proliferate in *in vitro* culture. Transduced Tu-57 (subculture C5, passage 5, 8 weeks in culture) was cultured in dox+ medium, fixed and subjected to immunofluorescent staining with **(A)** anti-c-MYC (yellow, nuclear), **(B)** the chromaffin cell marker anti-NSE (blue, cytoplasmic) and **(C)** a marker for dividing cells, EdU (red, nuclear), added 24 hours prior to fixation. **(D)** The three stainings in overlay. **(E)** Colour-enhanced and annotated version of Fig. 5D shows numerous NSE+ (tumour) cells that have undergone cell division during the previous 24hr Edu staining period (blue cytoplasm with a red (EdU) or orange (EdU+c-MYC) nucleus. Ovals highlight some examples). **(F)** Quantification of **(D)** . Experiments conducted in duplicate.

To provide further evidence of PPGL tumour cell proliferation, we stained the Tu57 C5 (passage 5) subculture ( Figs. 5A–D ) with EdU (24 hrs), together with c-MYC and neuron-specific enolase as a marker for chromaffin cells ( Figs. 5D & E ). Quantification ( Fig. 5F ) showed that around 30% of cells were NSE+ chromaffin cells and around half of these (NSE+ Edu+ 16%) underwent cell division in the previous 24hrs.

## Discussion

In this study we describe the first observation of oncogene-driven human paraganglioma tumour cell proliferation in cell culture. c-MYC transduction successfully induces proliferation of human paraganglioma cells in vitro and results in tumour subcultures predominantly consisting of cells expressing the tumour markers synaptophysin or NSE, even in later passage cultures. This was in stark contrast to our earlier study, in which we found that synaptophysin+ tumour cells largely disappear from paraganglioma and adrenal paraganglioma cultures over a 4–8 week period 23 . This study presents preliminary findings, focused on only two individual tumours, so further investigation is needed. Nonetheless, we show that primary chromaffin cells can be successfully transduced, suggesting that other oncogenes could also be considered. As we did not isolate chromaffin cells prior to transduction, this might have enhanced our results. No methods have been described to achieve this, but initial experiments not described here suggested that CD56 (NCAM) might be a suitable cell-surface marker for FACS-based isolation of chromaffin cells from dispersed paraganglioma cell cultures.

### Properties of a PPGL cell line

We have reviewed the history, as well as the pros and cons, of PPGL cell model systems elsewhere 22 . It can be argued that any paraganglioma-adrenal paraganglioma tumour cell line would have limited utility if it retains the growth properties generally displayed by these tumours in vivo. With a very low rate of growth, these tumours presumably exist in a semi-quiescent state with little cell cycling or DNA replication. This contrasts with many cancers that show robust growth and/or specific growth factor dependencies which can be utilized to kill tumour cells while sparing normal cells. In the case of paragangliomas this well-proven strategy appears problematic, and the Achilles heel of these tumours is unlikely to be found in the inhibition of cell growth. This makes the acquisition and analysis of a relevant human tumour cell model all the more important. Alternative strategies for model development such as spontaneously proliferating 2D cultured cells, 3D organoids or induction via radiation and growth factors ( 23 and personal communications) have been disappointing to date. We therefore propose that reproducible experimentation with human paraganglioma or adrenal paraganglioma cells will require a reliably inducible transgene that can turn cell proliferation on and off as required. This will allow maintenance and expansion of tumour cell numbers prior to experimentation, and following withdrawal of induction (e. g., doxycycline), the study of tumour cells in their naturally indolent state.

### Study strengths and limitations

In this study we describe sustained in vitro chromaffin tumour cell proliferation. Nonetheless, the study has a number of limitations, perhaps the most important being that Tu57 clones ceased proliferating around passage 18 for unknown reasons. Whether earlier frozen clones of Tu55 or Tu57 have the potential to sustain proliferation over a longer period was not investigated.

The identity of tumour-derived chromaffin cells was determined via expression of validated chromaffin cell markers synaptophysin, neuron-specific enolase or chromogranin A. Additional characterizations such as via alternative cell markers, RNAseq, as well as well-known molecular cellular signatures of SDH dysfunction 19 29 30 were not investigated.

In some experiments the detectable proportion of c-MYC+ cells was lower than the proportion of synaptophysin+ cells. As spontaneous growth of chromaffin tumour cells is unlikely 23 , some other explanation must be sought. One explanation is limited sensitivity of immunocytochemistry, with c-MYC protein levels below the detection threshold in some cells but still sufficient to induce proliferation. Speculatively, tumour cells may limit c-MYC expression to levels conducive to survival and proliferation. The impact of c-MYC or any other introduced oncogene on cellular processes could be investigated in detail using modern molecular approaches, which would help to validate and reassure users of these models. We note that a similar analysis has never been reported for any of the existing radiation-dependent models 23 .

## Conclusion

This pilot study strongly suggests that the hitherto neglected strategy of oncogene transduction 22 is feasible in primary human paraganglioma-adrenal paraganglioma tumour cells. The transduction of these cells with a proliferation-inducing transgene triggers cell division and enhances cell survival. The experimental approach outlined here may therefore offer a viable pathway to an experimental system of SDH-related human disease unique in a field with few relevant, validated models of any kind.

## Ethics Statement

This study was approved by the Medical Ethics Commission of Leiden university Medical Center (P12.082). The surgical material used in this study was provided and used entirely anonymously.

## Supplementary Material

Supplementary Material Zusatzmaterial
