# Supplementary material for: Pilot study: transduction of primary paraganglioma chromaffin tumour cells with inducible c-MYC drives cell proliferation
Source: Exp Clin Endocrinol Diabetes. 2026 Mar 25;134(3):71–8. doi: 10.1055/a-2797-3576 (PMC13016827; doi:10.1055/a-2797-3576)
Supplement: Supplementary file 1 — Supplementary Material [file 10-1055-a-2797-3576-08-2025-0234-endo.pdf]

Appendix 1. Materials and methods for the study: Pilot study: transduction of primary paraganglioma chromaffin cells with inducible MYC drives cell proliferation. Zhang et al.

Surgical material

Primary tumour tissue was obtained from irreversibly anonymized donors as surgical waste material. This study was therefore issued with a waiver by the Leiden University Medical Ethics Committee (CME protocol P12.082). Information on the location of the tumour and the gene involved (if known) was provided by the pathologist and clinical genetic services. Material was retrieved from the pathology department as soon as possible after surgical removal, usually within 2 hours, and placed in serum-free medium (DMEM/F-12 with GlutaMAX, 31331-028, GIBCO Thermo Fisher Scientific). Tissue was first minced and then digested with sterilized collagenase-dispase (Sigma Aldrich-Roche, 10 269 638 001) in Hanks' Balanced Salt Solution (HBSS) with Ca^{2+}/Mg^{2+} (GIBCO Thermo Fisher Scientific, 14065-049). The tumours were then processed as described [11].

Cell culture

Culture of human primary paragangliomas and pheochromocytomas has been described extensively in Bayley et al. 2022 [11]; Briefly, primary tumour tissue was initially cultured in polystyrene 6-well plates (Greiner Bio-One CellStar, Sigma-Aldrich) using 5% Foetal Bovine Serum (FBS) medium (DMEM/F-12 (Thermo Fisher 31331), 5% FBS, 1x Insulin-Transferrin-Selenium-Sodium Pyruvate (ITS-A; Thermo Fisher 51300044 and Penicillin-Streptomycin (P/S; Thermo Fisher 15070063)) prior to transduction. Cultures were then transferred to 50 ml cell culture flasks when approaching confluency (Greiner Bio-One CellStar, Sigma-Aldrich). All liquids were warmed in a 37°C water bath prior to use. Passaging of cultures was carried out by either briefly rinsing with 1x phosphate-buffered saline (PBS), followed by the addition of 0.5 ml trypsin-EDTA (Thermo Fisher 15400054) and incubation for around 2 minutes at 37°C until all cells detached, or by vigorous trituration in culture media, followed by centrifugation at 20 g for 5 minutes to pellet cells. The cell pellet was then

resuspended in 5 ml of fresh medium, transferred to a sterile 50 ml culture flask and returned to a 37°C (95% air, 5% CO_2) incubator. Media were refreshed weekly or bi-weekly.

Primary tumour cells and cell lines

The primary paraganglioma tumour cultures Tu47/Tu55/Tu57 were studied. Tu47 and Tu57 were carotid body PGLs carrying the SDHD Dutch founder variant, p.(Asp92Tyr), while Tu55 was a carotid body PGL carrying the SDHB variant p.(Arg217Cys). In the case of Tu47, the tumour culture had been maintained for around 6 months prior to transduction so likely consisted of predominately tumour-derived fibroblasts rather than actual chromaffin tumour cells. Immunocharacterization of cytospins (see below) found 50-60% smooth-muscle actin+, 10% Ki-67+, >1% synaptophysin+ and 0% MYC+ cells. Cells were cultured at 37°C in a humid 95% air/5% CO_2 incubator (Thermo Electron Corporation, NY, USA), all media and reagents were pre-warmed to 37°C, and cells were passaged approximately twice weekly depending on the confluency of the cells. To passage cells, medium was first removed, followed by a brief, gentle rinse with PBS, after which 1x trypsin-EDTA was added and the cells were incubated for 2 minutes at 37°C. When detached, the cells were harvested in appropriate medium and transferred to a new culture vessel and returned to the 37°C incubator. Tu47, Tu55, and Tu57 were cultured in DMEM/F12, 5% FBS, 1x ITS-A and P/S. Lentiviral-infected primary cells were exposed to 100 ng/ml doxycycline hyclate (Sigma-Aldrich, USA, D9891) in primary tumour cell medium. HEK293T cells and the neuroblastoma cell line, SH-SY5Y, were used in lentiviral production and as a source of conditioned medium, respectively. The HEK293T cell line was cultured in DMEM high glucose (4.5 g/L) & GlutaMAX & L-pyruvate (Thermo Fisher 31966), 10% FBS (Gibco, 10270098) and penicillin/streptomycin (P/S) (Thermo Fisher, 15140148). The SH-SY5Y neuroblastoma cell line was cultured in DMEM/F12 (Thermo Fisher 31331), 10% FBS and P/S.

Lentiviral production

As a transduction system, lentivirus has a number of advantages including stable genome integration, the ability to efficiently transduce a range of mammalian cell types, and especially

important in the case of PGL tumour cells, the ability to transduce non-dividing cells. Up to five different lentiviruses can also be transduced concurrently. To allow controlled induction of MYC expression, cells were transduced with a lentivirus carrying a Tet regulation system. Tet-dependent regulation of gene expression is based on a tetracycline-response-element (TRE) that consists of seven repeats of a 19-nucleotide tetracycline-operator sequence (tetO). The tetO repeat sequence is recognized by the tetracycline-repressor (tetR) protein. Originally designed as a transcriptional repression system (Tet-Off) requiring tetracycline to bind and release tetR from tetO, thus increasing gene expression, in 1995 Gossen and colleagues [16] identified and mutated tetR amino acid residues, generating a tetR protein that binds and activates tetO only in the presence of tetracycline. This so-called reverse tetracycline-controlled transactivator (rtTA) is a tetracycline-on (Tet-On) system, with induction of gene expression following administration of the compounds tetracycline or the more commonly used doxycycline (Dox) that interact with the tetR protein. Doxycycline was used in all experiments in this study.

To generate lentivirus, human embryonic kidney HEK293T cells were transfected with a pLP/VSVG plasmid, a lentiviral packaging plasmid expressing the vesicular stomatitis virus G glycoprotein (a gift from Dr Manuel Goncalves), a psPAX2 plasmid, a second-generation lentiviral packaging plasmid including HIV-1 gap and pol sequences (a gift from Didier Trono, Addgene plasmid # 12260), and either a human MYC^{T58A} construct (FU-tet-o-hMyc plasmid, a gift from Konrad Hochedlinger, Addgene #19775) or an rtTA plasmid (FUdeltaGW-rtTA, a gift from Konrad Hochedlinger, Addgene #19780) carrying a reverse tetracycline transactivator sequence [17]. The lentiviral plasmids, together with either MYC or rtTA, allow HEK293T cells to produce lentivirus in the culture media carrying either MYC or rtTA. A GFP-expressing lentiviral vector was transfected in parallel as a positive control for transfection efficiency. To ensure that the MYC construct used in experiments had no additional mutations beyond the stabilizing T58A variant, the nucleotide sequence was determined by sequencing. No additional variants were found.

Transfection and transduction

One day prior to transfection, HEK293T cells were seeded in a 10 cm diameter Petri dish (cell density of 70%-90%). The following day, 3.8 µg psPAX2, 2.1 µg pLP/VSVG, and 7.0 µg of MYC or rtTA plasmid were mixed with Lipofectamine 2000 (Invitrogen, USA) and serum-free Opti-Mem medium (Invitrogen, USA) in a sterile Eppendorf tube and incubated at room temperature for 20 minutes to allow DNA-Lipofectamine 2000 to combine. Lipofectamine 2000 was used at a ratio of 3:1 to total DNA. The DNA-Lipofectamine 2000 complexes were mixed by pipetting and then gently added to a 10 cm Petri dish containing HEK293T cells. Cells were incubated at 37°C overnight. Cell media were refreshed the following morning and the culture was further incubated to allow expression, maturation and release of mature virus. Two days after transfection, the lentiviral supernatants were collected and stored at -80 degrees.

To establish whether cells derived from a primary tumour culture (Tu47, a carotid body tumour carrying the SDHD variant p.(Asp92Tyr)) could be successfully transduced with lentiviruses containing MYC and rtTA and to establish the optimal ratio of MYC to rtTA, we transduced the culture with lentiviruses in several different ratios. Lentivirus (see above) was added to primary cells in a 1:1, 1:2 or 1:3 ratio (MYC:rtTA) together with polybrene (TR-1003-G , Sigma-Aldrich, ST Louis, USA). Cells were then incubated with virus for 24 hours at 37°C at which point fresh medium (DMEM + 5% FBS) was added. On the second day after transduction fresh medium was added, with or without 100 ng/mL doxycycline, and the transduced cells were further cultured on chamber slides. After 11 days of culture of both doxycycline-positive and negative cultures (+ and -), cells were fixed, processed and analysed for expression of MYC. In later transductions, lentivirus supernatants were added at a 1:3 ratio (MYC:rtTA) to a 12-well plate containing primary tumour cells together with polybrene.

Subcloning

Fluorescence-activated cell sorting (FACS) subcloning of transduced cells was aided by use of conditioned medium (CM) derived from SH-SY5Y and Tu47, filtered before use to remove remaining

cells and debris. Prior to FACS of cells, round-bottom 96-well plates were prepared with a 1:1 mix of conditioned and fresh medium, supplemented with 10 ng/ml doxycycline and cocktails (bathocuprione disulphonate and α-thioglycerol). Transduced primary tumour cells were trypsinized for 2 minutes at 37°C, Hank's solution (Gibco, California, USA) was then added, and cells were centrifuged at 50 g for 3 minutes. The cell pellet was resuspended in Hanks' solution containing 1% FBS, cells were counted and Hanks'/1% FBS was added to achieve a dilution of 1.0 x 10^6 cells/ml. Thirty minutes prior to flow sorting, propidium iodide (Thermo fisher, NY, USA. P3566) was added to the cell suspension at a final concentration of 1 µM and cells were kept on ice until sorting. Sorting of single cells into individual wells was carried out using a BD FACSAria III machine (BD Bioscience).

Immunocytochemistry

Immunocytochemical analysis of primary or transduced PGL cultures was carried out using 8-chamber slides (154534, Thermo Fisher Scientific) or cytospins. Chamber slides consist of a 25 x 75 mm glass microscope slide with an attached 8-well polystyrene culture chamber, which were first coated with poly-D-lysine hydrobromide (1 mg/ml, P7405, Sigma-Aldrich) to improve adhesion. Each chamber division was filled with 200 µl medium and cells, and then returned to a 37°C (95% air, 5% CO_2) incubator. To allow reattachment of cells, transduced tumour cell cultures were maintained for a further 3-4 days before analysis.

A cell suspension of 0.5-1.0 x 10^6 cells/ml was used to prepare tumour cell cytospins. A filter card (VWR, Amsterdam, Netherlands) was placed on a Superfrost-plus slide and together inserted into a cuvette in the rotor of a Shandon cytospin centrifuge. The slides were first rinsed with 0.1% PBS/bovine serum albumin (BSA) at 1850 rpm, the cuvette was emptied, and the cell suspension added. The slides were then centrifuged again at 1850 rpm, removed and processed as below.

Prior to immunocytochemistry (ICC), chamber slides were processed as follows: culture medium was removed, cells were washed in Ca^{2+} and Mg^{2+} supplemented phosphate-buffered saline (PBS), PBS was removed and slides were dried in a flow cabinet for 1 hour. The chamber slides were first fixed with PBS-buffered 4% formaldehyde (Klinipath, Duiven, Netherlands, 4074.9001). Slides were then

subjected to antigen retrieval in 10 mM citrate buffer (pH 6.0) at 110 °C in an autoclave for 5 min., followed by passive cooling to 37°C and brief washing in PBS. In cases where peroxide techniques were used, endogenous peroxide was blocked by a 20 min incubation in methanol/H_2O_2 (98.5/1.5) (v/v) solution. Then the slides were washed and blocked twice in PBS/Tween 0.05%. Prior to immunocytochemistry, the slides were pre-incubated in PBS/5% skimmed milk (Scharlau, Barcelona, Spain) for 1 hour and then incubated overnight with the first antibody diluted in PBS/1% skimmed milk. The next day, slides were again washed in PBS/Tween 0.05% and incubated with BrightVision Poly-HRP (ImmunoLogic, Duiven, Netherlands, 100216) for ICC or with an alternative secondary antibody if used for immunofluorescence (IF). After another wash step in PBS/Tween 0.05%, ICC slides were exposed to DAB (Dako, California, USA, 10117111) for exactly 7 minutes. After washing in Milli-Q, slides were then briefly counterstained with haematoxylin (Klinipath, Duiven, Netherlands) and directly rinsed in running water. ICC slides were mounted with Kaiser's glycerol gelatine (Merck KGaA, Darmstadt, Germany) and IF slides with Vectashield (Vectorlabs), followed by sealing with clear nail polish.

Antibodies

Transduced primary tumour cultures were analysed with a range of antibodies that detect PGL cell-specific markers [11]. The following individual markers were used to detect chromaffin (chief/type I) tumour cells: synaptophysin (anti-synaptophysin; NCL-L-SYNAP-299, clone 27G12, Leica Biosystems, 1:1000), neuron-specific enolase (anti-neuron-specific enolase; M0873, Clone BBS/NC/VI-H14, DAKO, 1:2000) and chromogranin A (anti-chromogranin A; M0869, Clone DAK-A3, DAKO, 1:1000). Sustentacular (type II) cells were detected using anti-Glial Fibrillary Acidic Protein A (M0761, Clone 6F2, DAKO, 1:1000). Anti-smooth muscle actin (M0851, Clone 1A4, DAKO, 1:100) was used to estimate connective tissue cells. MYC was detected using Abcam, ab32072, clone Y69, 1:500. As a Western blot loading reference, we used mouse anti-alpha Tubulin monoclonal (T5168, clone B-5-1-2, Sigma-Aldrich, 1:2000). Anti-Ki-67 (M7240, Clone MIB-1, DAKO, 1:1000) was used as a marker for proliferating cells, and anti-IgG1 (X0931, DAKO, 1:100) and anti-IgG2b (X0944, Clone DAK-GO9,

DAKO, 1:100) were used as negative controls. Secondary antibodies were either Bright Vision Poly-HRP (DPVO55HRP, ImmunoLogic, Duiven, Netherlands), an anti-mouse/rabbit/rat IgG for peroxidase ICC, Goat anti Mouse-IR Dye 680 (926-68020, LI-COR Biotechnology USA, 1: 20,000) and Goat anti Rabbit-IR dye 800 CW (926-32211, LI-COR Biotechnology USA, 1:20,000) for Western blot, or Goat anti Mouse (IgG)-Alexa488 (1:400) and Goat anti Rabbit (IgG)-Alexa594 (1:400) for immunofluorescence applications.

Monitoring cell proliferation

Cell proliferation can be monitored using immunohistochemical staining for the protein Ki-67 (a widely-used marker protein that is expressed only during the cell cycle) as described above, or with 5-ethynyl-2'-deoxyuridine (EdU), a synthetic nucleoside analogue of thymidine that is incorporated into DNA during active DNA synthesis. Edu was used in accordance with the manufacturer's protocol (Click-iT EdU Alexa Fluor 555 kit, Invitrogen, California, USA, C10352, 2010 08 04). All positive cells on a chamber slide were counted visually by two observers, with cell numbers varying between 25 and 250 per chamber slide. Slide were prepared in dublo or triplo.

DNA sequencing

The DNA profile for tumour 55 [SDHB variant p.(Arg217Cys)] was provided by Dr. Charlotte J. Dommering (Dept Human Genetics, Amsterdam UMC, the Netherlands). For other tumours, DNA extraction from tissue was carried using DNAeasy kits (QIAGEN Benelux B.V. Venlo The Netherlands). The *SDHD* gene was amplified by PCR using standard procedures, and primer sequences are available on request (purchased from Sigma-Genosys, UK). Sanger sequencing was analyzed using Mutation Surveyor (Softgenetics, State College PA, USA). Mutations are described in accordance with the recommendations of the Human Genome Variation Society (HGVS).

Statistics

Differences between experimental outcomes were evaluated using Student's T Test in Graphpad (Graphpad Inc., San Diego, CA, USA) where appropriate.
